# Reliability-based numerical analysis of glulam beams reinforced by CFRP plate

**DOI:** 10.1038/s41598-022-17751-6

**Published:** 2022-08-10

**Authors:** Harrach Dániel, Muayad Habashneh, Majid Movahedi Rad

**Affiliations:** Department of Structural and Geotechnical Engineering, Széchenyi István University, Gyor, 9026 Hungary

**Keywords:** Engineering, Civil engineering

## Abstract

Most existed researches consider deterministic numerical analysis when dealing with structural models. However, the test results reveal that uncertainties are existing in most cases regarding some considerations such as material randomness and the lack of experience. Therefore, proposing a probabilistic design models have got attention of researchers according to its important role in predicting accurate performance of the structures. The aim of the proposed work is to consider reliability-based analysis in numerical modelling of glulam beams reinforced with CFRP plates as well as unreinforced glulam beams by considering the properties of used timber material as random variables having mean value and standard deviation taking into consideration that the findings of this study have shown that the reliability index is worked efficiently as a limit which controls the process. Hill yield criterion model is adopted with respect to the data which is obtained from the experimental tests in order to validate the models. Furthermore, a detailed comparison between the reinforced and unreinforced glulam beams are proposed to see the effect of introducing the CFRP plates as a reinforcement material. The results of this study have successfully given a deep understanding of how the uncertainties plays a crucial role on the resulted deformations and stresses in which it was founded by making a comparison between deterministic and probabilistic numerical analysis.

## Introduction

Using timber as a construction material is one of the oldest techniques in structural engineering projects, especially in the case of structures carrying high self-weight since it has a relatively high strength to weight ratio, also it can be considered a highly sustainable material. During the last decades, growing interest in the application of timber in construction projects because of its ability to resist dynamic loads and its mechanical characteristics, thus many research works are involved^1–5^.

At the present time, various structural wood products are exhibiting a strong growth, glue laminated timber (Glulam) is one of those products which is considered as one of the highest performance composite construction materials^6^. These designed items are comprised of different layers of dimensional wood that are attached together using high strength glue material to produce a single unit. Taking into consideration that this process reduces the natural growth such as knots in the timber material^7–9^.

In fact, the topic of glulam beams has attracted many researchers in the last decades which resulted in various experimental tests on such products. In the study of Anshari et al.^10^, compressed wood (CW) blocks were used to strengthen the glulam beams and the specimens were tested later, the study proved that using CW as a reinforcement material is economically and environmentally effective. The feasibility of glued laminated timber beams was studied by Bourreau et al.^11^, the aim was to find the gluing factors which offer satisfaction behavior of glulam was done, where the results of delamination tests showed that the gluing parameters need to be adjusted depending on the wood species. Navaratnam et al.^12^ presented an experimental study for investigating the mechanical performance of glued-in-rod (GIR) embedded in glued-laminated timber (GLT) beam where the results of the pull-out test revealed that the failure occurred by the interface GIR to GLT slippage and shear bond delamination. An experimental study was done by Issa and Kmeid^13^ to reveal the influences of introducing reinforcement materials on the glued-laminated timber beams where it was found that reinforcement plays a major role in changing the failure mode from brittle to ductile and the load-carrying capacity of the reinforced beams was increased too. Another experimental study was carried out on glued laminated beams by Rescalvo et al.^14^, by considering carbon composites as reinforcement materials in which the study concluded that the type and the position of reinforcement directly affect the mechanical behavior of the entire element. Morin-Bernard et al.^15^ investigated the effects of the finger joint profile of the laminated hardwood beams on the tensile strength and it is suggested that the investigated species could be appropriate for the manufacture of GLT with high tensile strengths.

Furthermore, since the first use of timber as construction material, there are special kinds of composite materials that have been used recently in engineering wood products for purposes of reinforcing these products such as using carbon fiber reinforced polymer (CFRP), glass fiber-reinforced polymer (GFRP) and basalt fiber reinforced polymer (BFRP)^16–24^. Nadir et al.^23^ presented an experimental study of using CFRP composite to strengthen laminated timber beams. For purposes of predicting timber beams’ behavior, Kim and Harries^25^ presented a model of timber beams strengthened with CFRP sheets. A nonlinear finite element model was proposed and validated through experimental tests of timber beams strengthened with CFRP composites by Khelifa et al.^26^. Also, Khelifa and Celzard^27^ proposed a numerical approach to emulate the flexural behavior of CFRP timber beams. By using fiber-reinforced polymer plates to reinforce glued laminated timber beams, Raftery and Harte^28^ proposed an experimental study to investigate the impact of fiber-reinforced polymers on glulam beams. De Jesus et al.^29^ proposed experimental and numerical models to investigate the impact of the CFRP on the mechanical behavior of timber beams and its contribution to the prediction of failure. By analyzing the results of the experimental test, Timbolmas et al.^30^ compared the results and relations of tension and compression elasticity modulus between glulam beams with and without CFRP sheets. Glišović et al.^31^ showed in their study that the addition of CFRP plate into glulam beams increases their load carrying capacity by performing a comparison between glulam beams reinforced with CFRP plate and unreinforced glulam beams.

According to the literature, we can say that there are several advantages of using CFRP in the case of strengthening timber. CFRP is durable, easily stick to timber and they are low-density materials. Besides, when they are used on the tension side of timber element, a significant amount of tensile stress is transferred from timber under bending which is allowing the timber compression side to yield^32^.

To fulfill the fundamental goal of structural engineering of proposing a structure that satisfies the conditions of serviceability and safety with sensible cost, the designer should deal with the uncertainties which might be related to applied loads and material properties^33,34^. Thus, reliability-based design approaches have been introduced into deterministic designs of timber structures^35–38^. Bui et al.^39^ investigated the effect of vibration frequencies randomness for engineered wood products by adopting Monte Carlo Simulation. A probabilistic glulam model was proposed by Kandler and Füssl^40^ considering random stiffness for each lamination case. Also, Kandler et al.^41^ investigated the effect of considering random stiffness fluctuations on the performance of glulam. Load bearing capacity of glulam beams was considered to propose a probabilistic technique through Monte Carlo simulation in the study of Frink et al.^42^.

This study aims to explore the effect of introducing reliability-based design on the numerical analysis of reinforced glulam beams with CFRP plate and unreinforced glulam beams. In addition, four-point bending tests of the two considered beams models are considered and the results of these tests are discussed. To seek the expected goal, a written code is made to do the probabilistic analysis by assuming that the introduced reliability index plays as a bound when the timber properties are considered randomly. Moreover, Monte Carlo technique is utilized for purposes of determining reliability indices according to the statistics of the parameters of timber properties.

## Modelling the behavior of timber

Timber as a construction material has several characteristics that make it a proper choice for the construction of buildings. In fact, it is very anisotropic with various properties in different directions due to its formulation of oriented fibers. Also, when compression is parallelly applied to the grain, it generates stress which deforms the cells about their longitudinal axis. Hill yield criterion is applied for purposes of modeling timber since the timber is considered an elastic perfectly plastic material. The theory relies on the idea of a generalization of the Huber–Mises–Hencky in which there is an allowed connection between the material strength and the anisotropic directions. In case of utilizing this criterion with taking into consideration the isotropic hardening choice, the yielding formulation is given by^43^:

1 $$f\left(\sigma \right)=\sqrt{{\left(\sigma \right)}^{T}\cdot [M]\cdot \left(\sigma \right)}-{{\sigma }_{0}}^{\left({\overline{\varepsilon }}^{p}\right)},$$

where $\sigma$ is the stress state, ${\left(\sigma \right)}^{T}$ represents the transpose of stress state, [*M*] is the mass matrix, ${\sigma }_{0}$ stands for the reference yield stress, and ${\overline{\varepsilon }}^{p}$ represents the equivalent plastic strain. However, in case of considering it with the kinematic hardening choice, the yielding formulation is expressed by:

2 $$f\left(\sigma \right)=\sqrt{{\left(\left(\sigma \right)-\left(\alpha \right)\right)}^{T}\cdot \left[M\right]\cdot \left(\left(\sigma \right)-\left(\alpha \right)\right)}-{\sigma }_{0},$$

where α represents the vector of yield surface translation. The Hill yield stress potential of a coordinate system accompanying with anisotropy system is expressed as:

3 $$f\left(\sigma ,{\sigma }_{y}\right)=F{\left({\sigma }_{22}-{\sigma }_{33}\right)}^{2}+G{\left({\sigma }_{33}-{\sigma }_{11}\right)}^{2}+H{\left({\sigma }_{11}-{\sigma }_{22}\right)}^{2}+2L{{\sigma }_{23}}^{2}+2M{{\sigma }_{31}}^{2}+2N{{\sigma }_{12}}^{2}-{{\sigma }_{y}}^{2}=0,$$

where $N,M,F,H,L$ and $G$ are coefficients which are determined according to the material properties in several orientations.

4 $$N=\frac{3}{2\cdot {R}_{12}^{2}},$$

5 $$M=\frac{3}{2\cdot {R}_{13}^{2}},$$

6 $$F=\frac{1}{2}\cdot \left(\frac{1}{{R}_{22}^{2}}+\frac{1}{{R}_{33}^{2}}-\frac{1}{{R}_{11}^{2}}\right),$$

7 $$H=\frac{1}{2}\cdot \left(\frac{1}{{R}_{11}^{2}}+\frac{1}{{R}_{22}^{2}}-\frac{1}{{R}_{33}^{2}}\right),$$

8 $$L=\frac{3}{2\cdot {R}_{23}^{2}},$$

9 $$G=\frac{1}{2}\cdot \left(\frac{1}{{R}_{33}^{2}}+\frac{1}{{R}_{11}^{2}}-\frac{1}{{R}_{22}^{2}}\right),$$

where *R*_*i:j*_ stands for the anisotropic yield stress ratios.

In addition, processed wood, fiber composites, titanium alloys and zirconium alloys also can be modeled by adopting this criterion.

## Reliability-based design

In this study, the reliability-based design is utilized by calling the primary idea of reliability analysis. The failure criteria can be estimated by ${X}_{R} \le {X}_{S}$ in which ${X}_{R}$ stands for the non-negative limit for ${X}_{S}$ considering that ${X}_{S}$ and ${X}_{R}$ are two independent random variables with probabilistic density functions ${f}_{R} ({X}_{S})$ and ${f}_{R} ({X}_{R} )$, respectively. Accordingly, Eq. (10) is used to estimate the probability of failure (${P}_{f}$)^44^.

10 $${P}_{f}= P\left[{X}_{R}\le {X}_{S}\right]= {\iint }_{{X}_{R}\le {X}_{S}}{f}_{R} \left({X}_{R}\right){f}_{S} \left({X}_{S}\right)d{X}_{R}d{X}_{S}.$$

An alternative definition can be used for the previous equation in which it is defined in the matter of the limit state function:

11 $$g\left({X}_{R},{X}_{S}\right)={X}_{R}-{X}_{S},$$

where $g \le 0$ characterizes the domain of failure ${D}_{f}$. Thus, to obtain ${P}_{f},$ the following expression is used:

12 $${P}_{f}={F}_{g}\left(0\right).$$

In addition, ${P}_{f}$ could be written as:

13 $${P}_{f}={\int }_{g({X}_{R},{X}_{S})\le 0}f\left(X\right)dX={\int }_{{D}_{f}}f(X)dX.$$

A mathematical technique which is called Monte Carlo method is used in this study for the purposes of estimating the ${P}_{f}$. The primary idea of this method implicates the generating $x$ of the random vector $X$ based on the probability joint density function ${f}_{X}(x)$. According to the Monte-Carlo technique, the ${P}_{f}$ can be estimated as the ratio of points number within the failure domain to the total generated points number. The formulation which is used to express this hypothesis can be written by utilizing the indicator function of ${D}_{f}$ as:

14 $${\chi }_{{D}_{f}}\left(x\right)=\left\{ \begin{array}{c}1 \quad if \; x\in {D}_{f}\\ 0 \quad if \; x\notin {D}_{f}\end{array}\right\}.$$

Thus, ${P}_{f}$ formula can be reconstructed as:

15 $${P}_{f}={\int }_{-\infty }^{+\infty } . . . {\int }_{-\infty }^{+\infty }{\chi }_{{D}_{f}}\left(x\right){f}_{X}\left(x\right)dx.$$

Therefore, the two points distribution of the random variable ${\chi }_{{D}_{f}}\left(X\right)$:

16 $${\mathbb{P}}\left[ {\chi }_{{D}_{f}}\left(X\right)=1\right]= {P}_{f},$$

17 $${\mathbb{P}}\left[ {\chi }_{{D}_{f}}\left(X\right)=0\right]= {1-P}_{f},$$

where ${P}_{f}={\mathbb{P}}[X\in {D}_{f }]$. Considering that ${\chi }_{{D}_{f}}\left(X\right)$ is associated with mean value and variance which are determined by:

18 $${\mathbb{E}}\left[{\chi }_{{D}_{f}}\left(X\right)\right]=1\cdot {P}_{f}+0\cdot \left(1-{P}_{f}\right)={P}_{f},$$

19 $${\mathbb{V}}ar\left[{\chi }_{{D}_{f}}\left(X\right)\right]={\mathbb{E}}\left[{\chi }_{{D}_{f}}^{2}\left(X\right)\right]-({\mathbb{E}}\left[{\chi }_{{D}_{f}}\left(X\right)\right]{)}^{2}={P}_{f}-{P}_{f}^{2}={P}_{f}\left(1-{P}_{f}\right).$$

In Monte Carlo technique, an estimator of the mean value to determine ${P}_{f}$ is expressed as follow:

20 $$\widehat{\mathbb{E}}\left[{\chi }_{{D}_{f}}\left(X\right)\right]=\frac{1}{Z}\sum_{z=1}^{Z}{\chi }_{{D}_{f}}({X}^{(z)})={\widehat{P}}_{f},$$

where ${X}^{(z)}$ represents independent random vectors (where $z=1,\dots ,Z$) which are associated with probability density functions. For purposes of considering uncertainties, the material properties of timber beams are considered as random variables in which following Gaussian distribution with mean value ${\mathbb{E}}$ and variance ${\mathbb{V}}ar$. Consequently, the estimator’s mean value and variance are calculated as:

21 $${\mathbb{E}}\left[{\widehat{P}}_{f}\right]=\frac{1}{Z}\sum_{z=1}^{Z}{\mathbb{E}}\left[{\chi }_{{D}_{f}}\left({X}^{\left(z\right)}\right)\right]=\frac{1}{Z}Z{P}_{f}={P}_{f},$$

22 $${\mathbb{V}}ar\left[{\widehat{P}}_{f}\right]=\frac{1}{{Z}^{2}}\sum_{z=1}^{Z}{\mathbb{V}}ar\left[{\chi }_{{D}_{f}}\left({X}^{\left(z\right)}\right)\right]=\frac{1}{{Z}^{2}}Z{P}_{f}\left(1-{P}_{f}\right)=\frac{1}{Z}{P}_{f}\left(1-{P}_{f}\right).$$

Due to the difficulties in computing the probability of failure accurately in practical structures, first-order reliability methods are used where they utilize a measure known as reliability index which is denoted by the Greek letter beta (β)^45^. The advantages of using a reliability index are that as the reliability-based design has found wide applications in structural engineering, the target reliability index governs more everyday structural engineering practices and structural engineering standards offer a wide range of target values (see e.g., EN1990^46^).

The reliability bound can be demonstrated by considering the reliability index $\upbeta$ as:

23 $${\upbeta }_{\mathrm{target}}-{\upbeta }_{\mathrm{calc}}\le 0.$$

Finally, in order to determine ${\upbeta }_{\mathrm{target}}$ and ${\upbeta }_{\mathrm{calc}}$, the following equations are used:

24 $${\upbeta }_{\mathrm{target}}=-{\Phi }^{-1}\left({P}_{f,target}\right);$$

25 $${\upbeta }_{\mathrm{calc}}=-{\Phi }^{-1}\left({P}_{f,calc}\right).$$

## Experimental work

In this section, two experiments are considered in which the first one examines the unreinforced glulam beams while the second experiment represents the test of glulam beams which is reinforced by CFRP plate. The beams are tested by utilizing four-point bending tests^29^. An adhesion test was performed on the timber before the beginning of the test. Moreover, a commercial glulam beams are used in which the properties are illustrated by the producer. The considered properties of the used materials in this study are concluded in Table 1, where f_m,k_ is characteristic bending strength, f_c,0,k_ represents characteristic compression strength parallel the grain, f_c,90,k_ stands for characteristic compression strength perpendicular to the grain, f_t,0,k_ is characteristic tension strength parallel the grain, f_t,90,k_ represents characteristic tension strength perpendicular to the grain, f_v,k_ is characteristic shear strength, E_0,mean_ stands for mean of parallel the grain elastic modulus, and E_90,mean_ is mean of perpendicular to grain elastic modulus.

**Table 1:** Materials properties.

Material	Type	Flexural strength (N/mm^2^)	Compression strength (N/mm^2^)	Tensile strength (N/mm^2^)	Shear strength (N/mm^2^)	Elastic modulus (N/mm^2^)
Timber	GL24h	f_m,k_ = 50.0	f_c,0,k_ = 29.0 f_c,90,k_ = 3.2	f_t,0,k_ = 30.0 f_t,90,k_ = 0.4	f_v,k_ = 4.0	E_0,mean_ = 9400 E_90,mean_ = 390
CFRP	Carbodur	–	–	3100	–	170,000
Epoxy glue	Sikadur-30	–	85–95	26–31	16–19	9600

Six glulam beams were considered for the experimental tests, three of them were considered as unreinforced glulam beams each of them consisted of six layers, each layer was $40 \mathrm{mm}$ height. Thus, the geometry of single beam was $2500 \mathrm{mm}$ length and a cross-sectional area of $\left(100 \mathrm{mm} \times 240 \mathrm{mm}\right)$. The other three glulam beams which were considered for reinforcement had the same geometry, but pultruded CFRP plate was chosen as reinforcement material with dimensions of $2500 \mathrm{mm}$ in length, $100 \mathrm{mm}$ of width and $1.2 \mathrm{mm}$ thickness (Sika CarboDur S-1012). To verify its tensile strength and elastic modulus, the CFRP plate was tested in tension according to Ref.^47^. Tensile strength of 3100 MPa and a modulus of elasticity in the tension of 170,000 MPa were determined and confirmed by the manufacturer in the technical data report^48^. Besides, adhesives SIKA products were used to glue the CFRP plate. The schematic layout of the laboratory tests of unreinforced and reinforced glulam beams are illustrated in Figs. 2 and 6, respectively.

## Numerical modelling

In this section, FEA is proposed to model the nonlinear behavior of both reinforced and unreinforced glulam beams by using FEA software ABAQUS^49^.

### Unreinforced glulam beams

**Figure 1:**
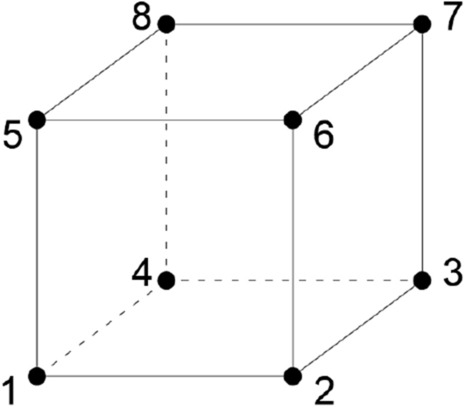
Eight-node brick element.

The unreinforced glulam beam is modelled by using C3D8 elements which are an eight-node brick elements as can be seen in Fig. 1. As lamellas are glued together, a perfect bond is assumed between these lamellas, and it was not considered in the model due to its small thickness. It is worth mentioning that for purposes of distinguishing between the compressive strength and tensile strength, a theoretical separation was proposed of compression and tension zones^50,51^. Besides, steel bearing plates are used at loading points in order to prevent the model from local failure and the dimensions of these plates are length $=150 \mathrm{mm}$, thickness = $30 \mathrm{mm}$ and width = $100 \mathrm{mm}$.

**Figure 2:**
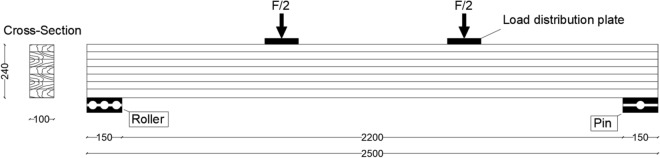
Geometry and the boundary conditions of the unreinforced glulam beam.

The geometry and the boundary conditions of the considered beams are presented in Fig. 2. Taking into consideration that because of symmetry, only the half of the beam is considered for the modelling. Besides, it should be noted that coupling effect are considered to distribute the loads on the plates.

**Figure 3:**
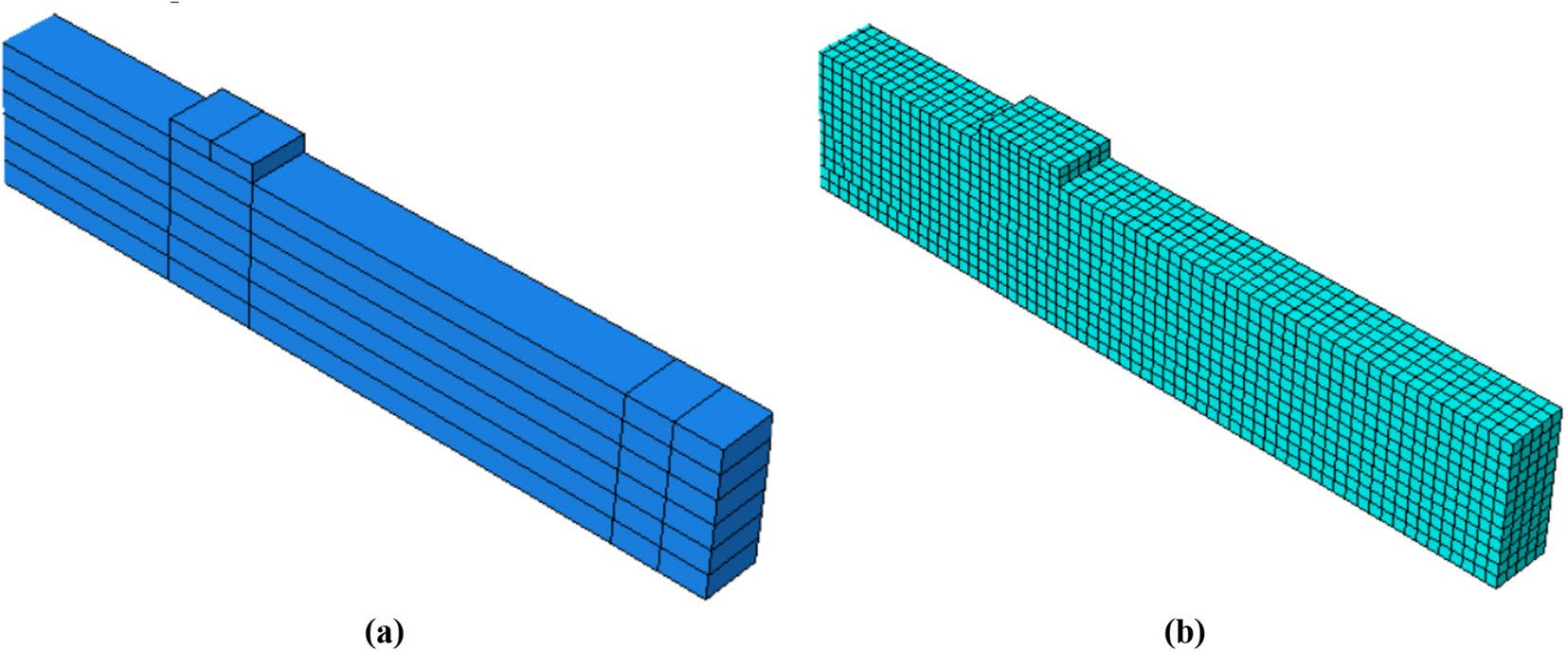
Unreinforced glulam numerical model: (**a**) Assembly of the model (**b**) Finite element mesh of the model.

Figure 3 represents the glulam model in ABAQUS, only half of the beam is considered while the deleted parts are replaced with proper symmetry constraints where approximately $32,000$ elements are used to generate a fine mesh of this half to produce accurate results.

The considered material properties of the model in FEA are shown in Tables 2 and 3 for compression and tension, respectively.

**Table 2:** Material properties—compression side.

Elasticity			Plasticity		
E_1_ = 9400 MPa	E_2_ = 0.53 GPa	E_3_ = 0.53 GPa	σ_yield_ = f_c,0,k_ = 29.0 MPa		
G_12_ = 0.72 GPa	G_13_ = 0.24 GPa	G_23_ = 0.2 GPa	R_11_ = 5.800	R_22_ = 0.640	R_33_ = 0.640
ν_12_ = 0.40	ν_13_ = 0.40	ν_23_ = 0.40	R_12_ = 1.386	R_13_ = 1.386	R_23_ = 1.386

**Table 3:** Material properties—tension side.

Elasticity			Plasticity		
E_1_ = 9400 MPa	E_2_ = 0.39 GPa	E_3_ = 0.39 GPa	σ_yield_ = f_t,0,k_ = 30.0 MPa		
G_12_ = 0.72 GPa	G_13_ = 0.24 GPa	G_23_ = 0.24 GPa	R_11_ = 6.000	R_22_ = 0.080	R_33_ = 0.080
ν_12_ = 0.40	ν_13_ = 0.40	ν_23_ = 0.40	R_12_ = 1.386	R_13_ = 1.386	R_23_ = 1.386

**Figure 4:**
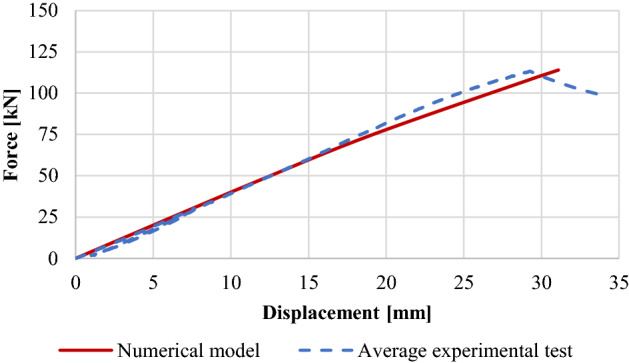
Force–displacement diagrams of the unreinforced glulam.

**Figure 5:**
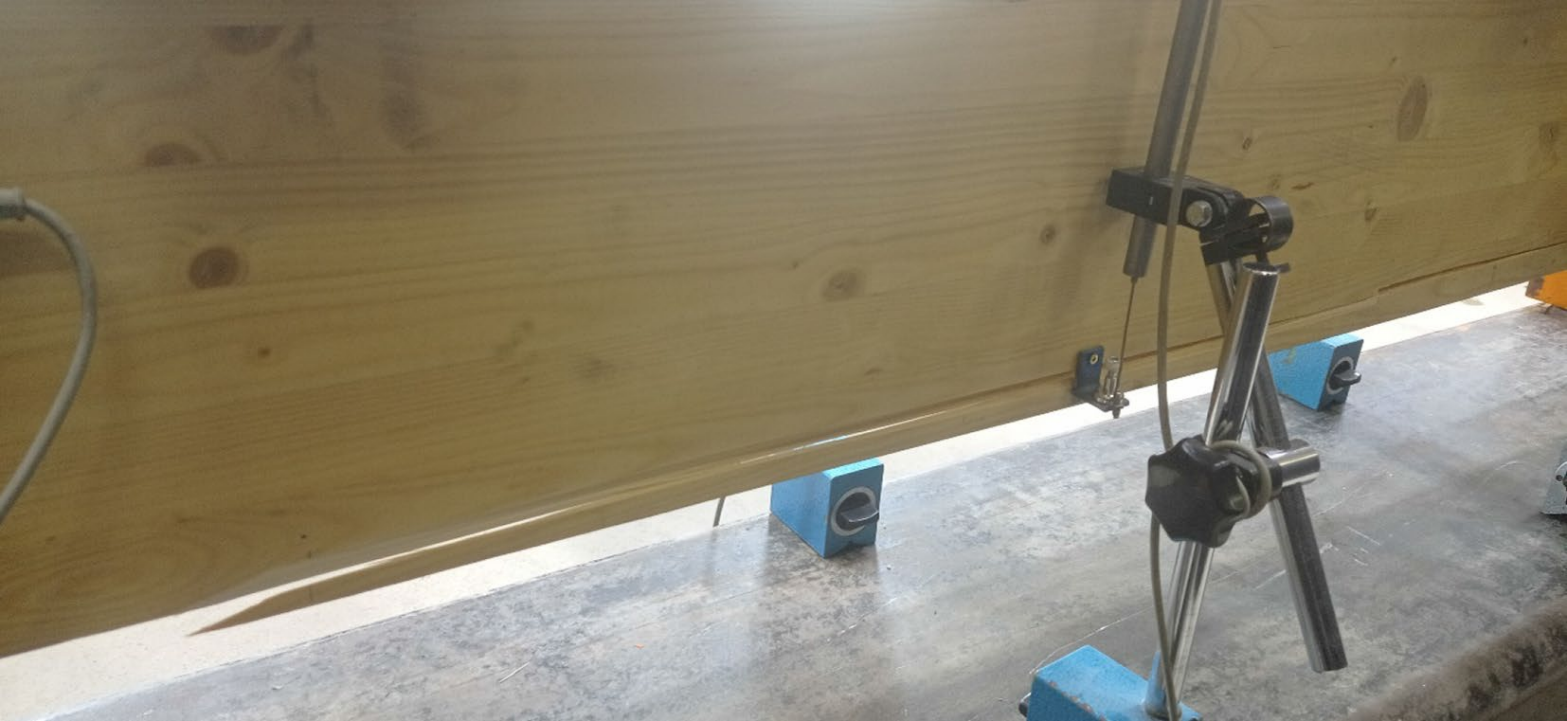
Failure mechanism of unreinforced beams.

Figure 4 represents a comparison between the validated model and the average experimental tests according to the obtained displacement at the middle of the models. In addition, tensile failure of unreinforced glulam beams is shown in Fig. 5 where the failure occurred within the region of maximum bending between the two acting loads where the tensile stresses exceed the yield strength. Taking into consideration that the adhesion between the laminates of the timber did not fail.

### Glulam beams reinforced with CFRP plate

**Figure 6:**
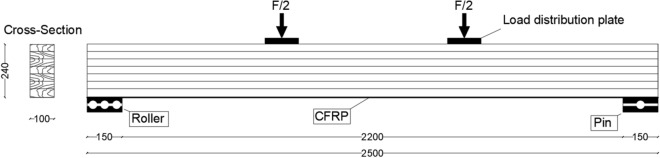
Geometry and the boundary conditions of the reinforced glulam beam.

The modelling of the glulam beam in this section is the same as what we did in the previous section but with difference which is represented in the introducing of the Sika CarboDur $\mathrm{S}-1012$ CFRP laminate with dimensions of length $=2500 \; \mathrm{ mm},$ width $=100 \; \mathrm{ mm}$ and thickness $=1.2 \; \mathrm{ mm}$ for the reinforcement of the glulam beams and Fig. 6 illustrates the geometry of the considered model. Also, just the half of the model is considered with FE mesh of $36,000$ elements.

**Figure 7:**
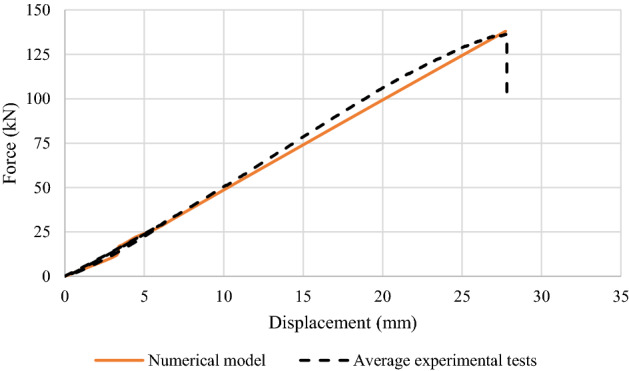
Force–displacement diagrams of the reinforced glulam.

**Figure 8:**
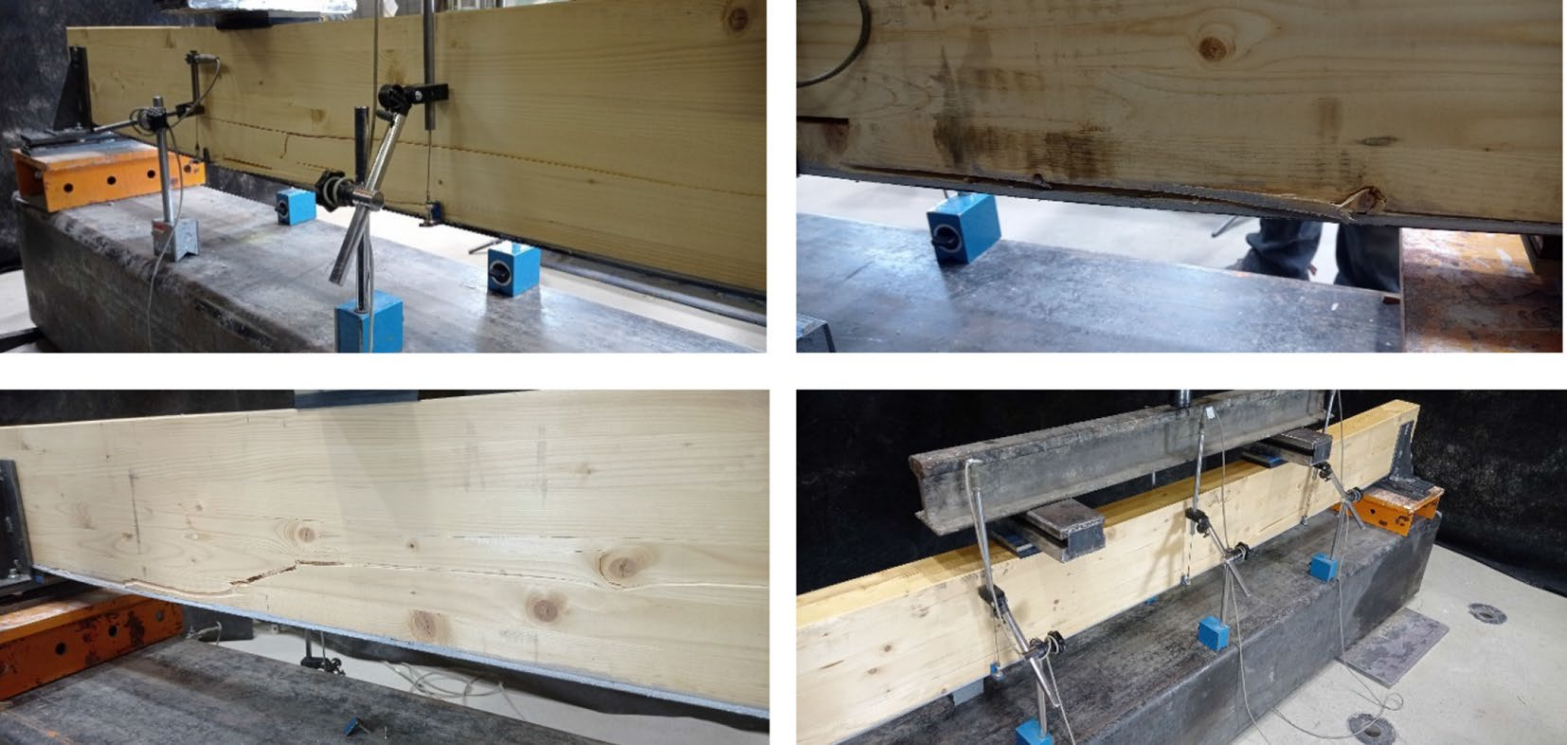
Failure mechanism of reinforced glulam beams.

The material properties of the considered model are the same as those which are shown in Tables 2 and 3. Besides, Fig. 7 shows the maximum obtained deflection at the middle point of the validated model in comparison to the average experimental tests. The force–displacement behavior was linear-elastic until the occurrence of the local fractures within tension zone. As compressive timber yielded, a nonlinear response was generated in which a sudden drop of load as a result of tensile failure in timber have been occurred as shown in Fig. 8. Besides, it is worth noting that there was no failure occurred within the CFRP plate.

## Results and discussion

In this section, a discussion about the obtained results of the unreinforced glulam beam and reinforced glulam beam with CFRP plate is considered as well as a detailed comparison between these results is taken into consideration too. As what was previously mentioned, the FEA software ABAQUS is used to validate the numerical models according to the collected data of the experimental tests. Then, a written code is made to do the probabilistic analysis by assuming that the introduced reliability index plays as a bound when the timber properties are considered as random variables with mean value and standard deviation. For purposes of calculating the reliability indices, Monte Carlo technique is adopted by assuming the total sample point number (Z = 3 × 10^6^). Furthermore, the assumed random variables of the timber material are shown in Table 4, taking into consideration that the corresponding parameters are changed accordingly.

**Table 4:** Considered random variables of timber properties.

Parameter	Mean value	Standard deviation
f_c,0,k_ (N/mm^2^)	29.00	5%
f_c,90,k_ (N/mm^2^)	3.20	
f_v.k_ (N/mm^2^)	4.00	
f_t.0.k_ (N/mm^2^)	30.00	
f_t,90,k_ (N/mm^2^)	0.40	
E_0_ (N/mm^2^)	9400	
E_90_ (N/mm^2^)	390	
G (GPa)	0.72	
ν	0.40	

### Unreinforced glulam beam

Three different results of the unreinforced glulam beam analysis are considered according to three different reliability index $(\upbeta )$ values which are shown in Table 5. It can be noticed that by introducing $\upbeta$, it has worked as a bound in which the changing of timber properties changes the load $(\mathrm{F})$ and corresponding displacement $(\mathrm{U})$. The displacement values are decreased by $5.64\mathrm{\%}$ from $23.39 \; \mathrm{ mm}$ in case of $\upbeta =3.32$ to $22.07$ $\mathrm{mm}$ when $\upbeta =4.83.$ Besides, considering low values of $\upbeta$ will involve greater loads, consequently greater values of displacements will be resulted also. So, supposing that the randomness of timber properties will lead to producing random properties per each iteration, thus this explains how uncertainty’s part is adapted in this study.

**Table 5:** Results of unreinforced glulam beam analysis.

$\upbeta$	f_c,0,k_	f_c,90,k_	f_v.k_	f_t.0.k_	f_t,90,k_	E_0_	E_90_	G	ν	U	F
	N/mm^2^	N/mm^2^	N/mm^2^	N/mm^2^	N/mm^2^	N/mm^2^	N/mm^2^	GPa		mm	kN
4.83	27.44	3.35	4.02	31.40	0.41	9308	384	0.71	0.42	22.07	88
4.28	30.19	3.42	3.78	30.06	0.38	8478	386	0.68	0.41	23.16	90
3.32	26.02	3.14	3.83	26.60	0.45	9271	411	0.71	0.39	23.39	92

Additionally, the probabilistic nature of timber properties which was represented in Table 6, indicated that introducing the standard deviation on these values changed the results accordingly where the material properties are straightforwardly influencing load $(\mathrm{F})$ and corresponding displacement $(\mathrm{U})$ values regarding the resulted $\upbeta$ values.

**Table 6:** Mean von mises stresses values according to probabilistic analysis of the unreinforced glulam beam.

$\upbeta$	F (kN)	Displacement (mm)	Mean von mises stress (MPa)
4.83	88	22.07	12.04
4.28	90	23.16	12.35
3.32	92	23.39	12.64

**Figure 9:**
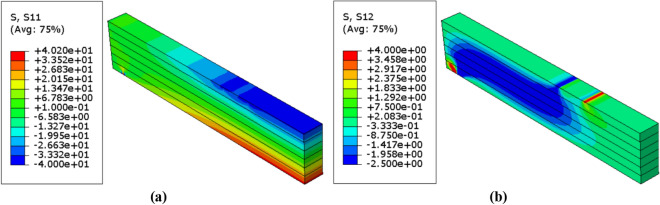
Stress distributions $(\mathrm{MPa})$ in the unreinforced glulam beam in case of $\upbeta =4.83$ (**a**) Normal stress ${\upsigma }_{11}$ (**b**) Shear stress ${\upsigma }_{12}$.

**Figure 10:**
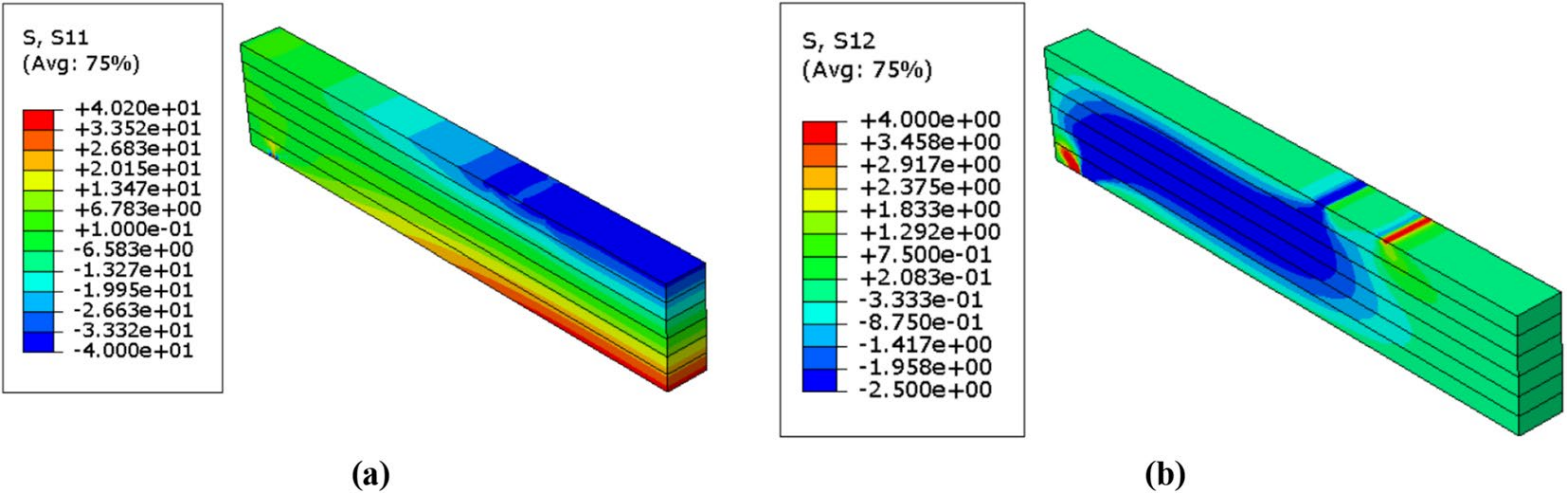
Stress distributions $(\mathrm{MPa})$ in the unreinforced glulam beam in case of $\upbeta =4.28$ (**a**) Normal stress ${\upsigma }_{11}$ (**b**) Shear stress ${\upsigma }_{12}$.

**Figure 11:**
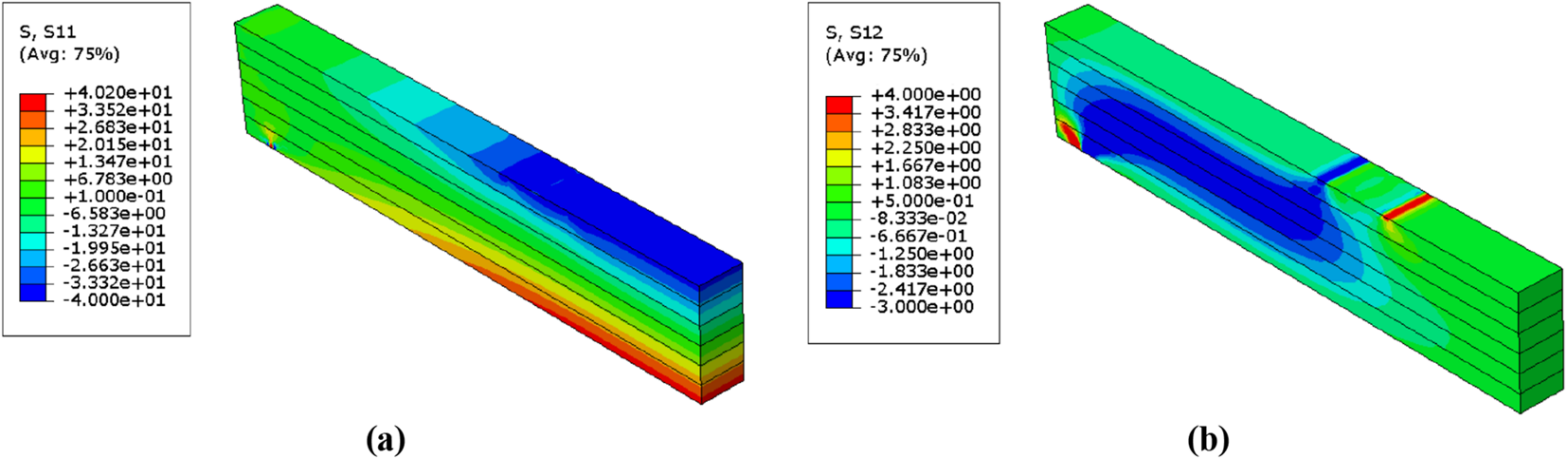
Stress distributions $(\mathrm{MPa})$ in the unreinforced glulam beam in case of $\upbeta =3.32$ (**a**) Normal stress ${\upsigma }_{11}$ (**b**) Shear stress ${\upsigma }_{12}$.

Due to symmetry, only half of the beam was considered to show the result of numerical analysis. The patterns of normal and shear stress distribution which are resulted from the probabilistic numerical analysis within the model are shown in Figs. 9, 10 and 11. Besides, Table 6 represents the corresponding mean von mises stresses, load, and displacement values in the probabilistic design for each value of $\upbeta$. The value of mean von mises stress is decreased by $4.75\mathrm{\%}$ from $12.64 \; \mathrm{ MPa}$ in case of $\upbeta =3.32$ to $12.04 \; \mathrm{ MPa}$ when $\upbeta =4.83$, thus we can say that as $\upbeta$ increases the mean von mises stress decreases.

**Figure 12:**
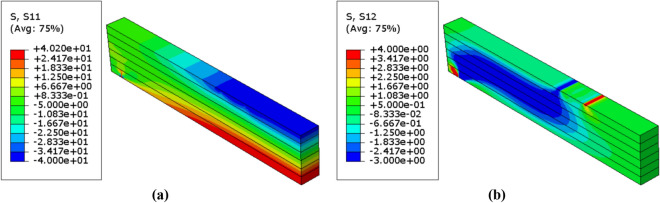
Stress distributions $(\mathrm{MPa})$ in the unreinforced glulam beam in case of deterministic analysis (**a**) Normal stress ${\upsigma }_{11}$ (**b**) Shear stress ${\upsigma }_{12}$.

On the other hand, the patterns of normal and shear stresses distribution in which are resulted from the deterministic numerical analysis within the model are presented in Fig. 12. Also, Table 7 represents the corresponding mean von mises stresses, load and displacement values in case of deterministic analysis.

**Table 7:** Stress distribution according to deterministic analysis of the unreinforced glulam beam.

F (kN)	Displacement (mm)	Mean von mises stress (MPa)
114	31.09	13.77

The value of mean von mises stress in the case of deterministic design is higher than what is obtained in the probabilistic design, thus we can understand that $\upbeta$ is working as a limit for producing safe design.

### Reinforced glulam beam with CFRP plate

The glulam beam with CFRP plate reinforcement is considered for the probabilistic analysis in this section, the obtained results corresponding to various $\upbeta$ values are shown in Table 8. By considering the reliability index, the corresponding load $(\mathrm{F})$ and displacement $(\mathrm{U})$ values are changed as the timber material properties are changed. For instance, the displacement values are decreased by $7.86\mathrm{\%}$ from $23.67\mathrm{ mm}$ in case of $\upbeta =3.32$ to $21.81$ $\mathrm{mm}$ when $\upbeta =4.83.$ Thus, again here we can say that the reliability index can be considered as a constraint in which new results are generated accordingly.

**Table 8:** Results of glulam beam reinforced with CFRP.

$\upbeta$	f_c,0,k_	f_c,90,k_	f_v.k_	f_t.0.k_	f_t,90,k_	E_0_	E_90_	G	ν	U	F
	N/mm^2^	N/mm^2^	N/mm^2^	N/mm^2^	N/mm^2^	N/mm^2^	N/mm^2^	GPa		mm	kN
4.83	29.72	3.35	3.82	29.73	0.36	9175	396	0.75	0.42	21.81	110
4.28	30.19	3.42	3.78	30.06	0.38	8478	386	0.68	0.41	22.10	116
3.32	33.05	3.14	3.61	33.05	0.38	9132	394	0.74	0.42	23.67	118

Similar to the findings of the previous problem, we can say here also that considering random variables of timber properties explains how the effect of introducing the $5\mathrm{\%}$ standard deviation is on these values changes the results accordingly where the material properties are straightforwardly influencing load $(\mathrm{F})$ and corresponding displacement $(\mathrm{U})$ values regarding to the obtained $\upbeta$ values.

**Figure 13:**
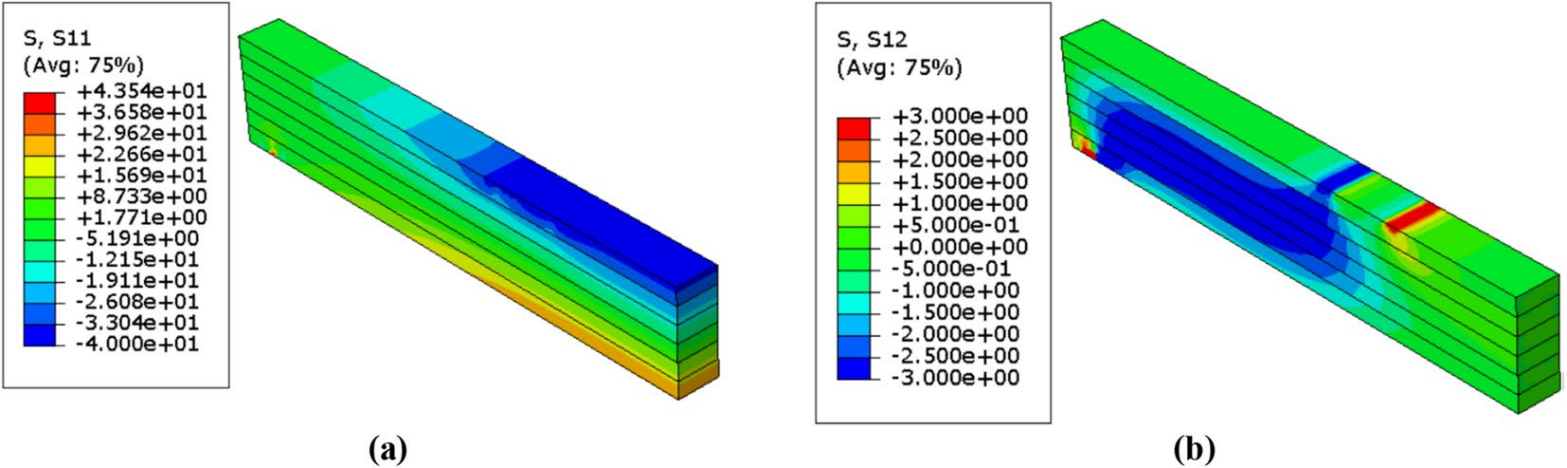
Stress distributions $(\mathrm{MPa})$ in the reinforced glulam beam in case of $\upbeta =4.83$ (**a**) Normal stress ${\upsigma }_{11}$ (**b**) Shear stress ${\upsigma }_{12}$.

**Figure 14:**
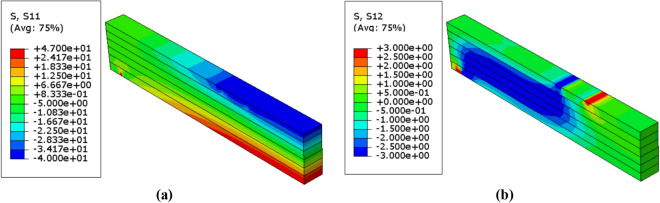
Stress distributions $(\mathrm{MPa})$ in the reinforced glulam beam in case of $\upbeta =4.28$ (**a**) Normal stress ${\upsigma }_{11}$ (**b**) Shear stress ${\upsigma }_{12}$.

**Figure 15:**
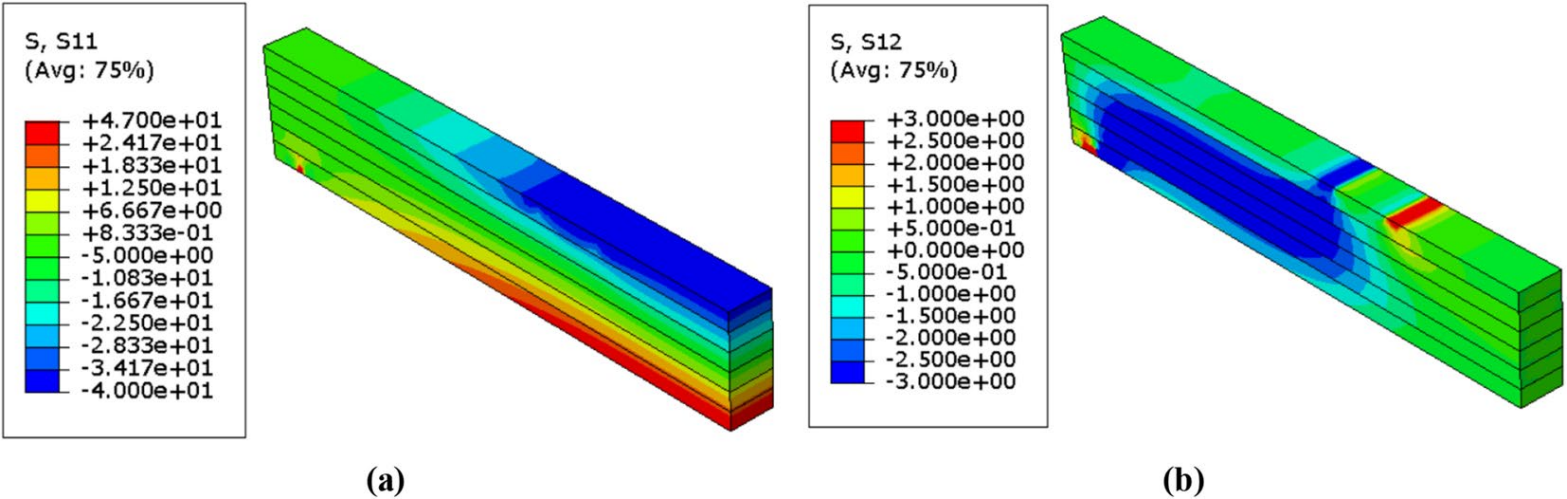
Stress distributions $(\mathrm{MPa})$ in the reinforced glulam beam in case of $\upbeta =3.32$ (**a**) Normal stress ${\upsigma }_{11}$ (**b**) Shear stress ${\upsigma }_{12}$.

The distribution of normal and shear stresses in which are resulted from the probabilistic numerical analysis of the reinforced glulam beam are presented in Figs. 13, 14 and 15. Taking into consideration that because of the symmetry of the beams, only the half of the beam was considered to express the result of analysis. Besides, Table 9 represents the corresponding mean von mises stresses, load and displacement values in the probabilistic design for each value of $\upbeta$. The value of mean von mises stress is decreased by $6.77\mathrm{\%}$ from $12.71 \; \mathrm{ MPa}$ in case of $\upbeta =3.32$ to $11.85 \; \mathrm{ MPa}$ when $\upbeta =4.83$, thus we can say that the mean von mises stress decreases as $\upbeta$ increases.

**Table 9:** Mean von mises stresses values according to probabilistic analysis of reinforced glulam beam.

$\upbeta$	F (kN)	Displacement (mm)	Mean von mises stress (MPa)
4.83	110	21.81	11.85
4.28	116	22.10	12.65
3.32	118	23.67	12.71

**Figure 16:**
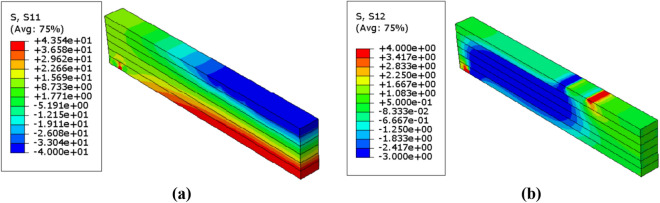
Stress distributions $(\mathrm{MPa})$ of the reinforced glulam beam in case of deterministic analysis (**a**) Normal stress ${\upsigma }_{11}$ (**b**) Shear stress ${\upsigma }_{12}$.

While Fig. 16 shows the normal and shear stresses distribution which are resulted from the deterministic numerical analysis of the reinforced glulam beam. Moreover, the corresponding mean von mises stresses, load and displacement values in case of deterministic analysis are presented in Table 10.

**Table 10:** Stress distribution according to deterministic analysis of reinforced glulam beam.

F (kN)	Displacement (mm)	Mean von mises stress (MPa)
138	27.74	14.72

In case of deterministic design , we can say that the calculated value of mean von mises stress is much higher than which is obtained from the probabilistic design. Therefore, $\upbeta$ efficiently works as a bound in order to produce safe design controlling the yielding state of the model.

### Comparison between reinforced and unreinforced glulam models

In this section, different comparisons between the obtained results of unreinforced glulam and reinforced glulam with CFRP are considered in order to show the effect of considering the CFRP plate as reinforcement material in case of probabilistic designs.

**Figure 17:**
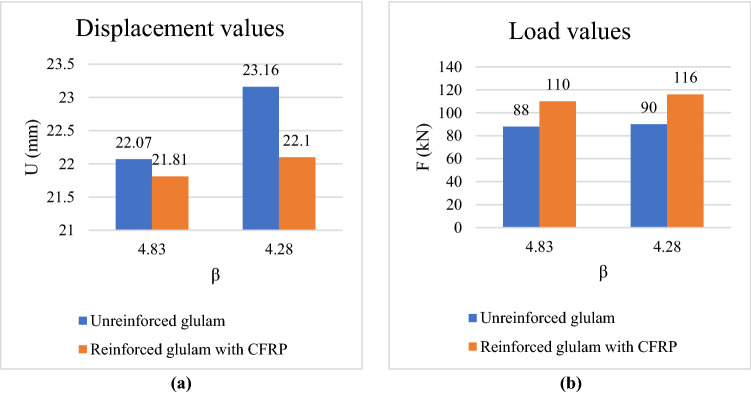
Obtained displacement and load values of the considered models.

A comparison between the obtained displacement of the two considered models according to different values of $\upbeta$ is represented in Fig. 17a. The displacement value is decreased by $1.17\mathrm{\%}$ from $22.07 \; \mathrm{ mm}$ in case of unreinforced glulam model to $21.81 \; \mathrm{ mm}$ in case of reinforced glulam model considering $\upbeta =4.83$. Besides, in case of $\upbeta =4.28$, displacement value is decreased by $4.58\mathrm{\%}$ from $23.16 \; \mathrm{ mm}$ in case of unreinforced glulam model to $22.1 \; \mathrm{ mm}$ in case of reinforced glulam model. Another comparison is made according to the obtained applied load values of the two considered models according to different values of $\upbeta$ is shown in Fig. 17b. The applied load value is increased by $20\mathrm{\%}$ in case of $\upbeta =4.83$ from $88 \; \mathrm{ kN}$ in case of unreinforced glulam model to $110 \; \mathrm{ kN}$ in case of reinforced glulam model. While the load value is increased by $22.41\mathrm{\%}$ in case of $\upbeta =4.83$ from $90 \; \mathrm{ kN}$ in case of unreinforced glulam model to $116 \; \mathrm{ kN}$ in case of reinforced glulam model.

**Figure 18:**
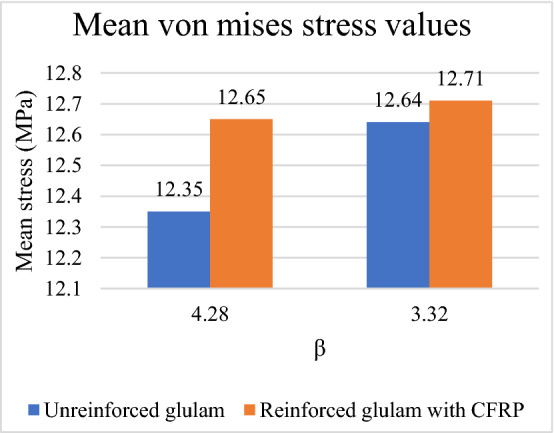
Mean von mises stress values of the considered models.

Also, a comparison is performed between the mean von mises stress values of the two considered models according to $\upbeta$ value which is seen in Fig. 18. It can be noticed that the mean von mises stress value is increased by $2.37\mathrm{\%}$ in case of $\upbeta =4.28$ from $12.35 \; \mathrm{ MPa}$ in case of unreinforced glulam model to $12.65$ $\mathrm{MPa}$ in case of reinforced glulam model. While the obtained mean von mises stress values are increased by $0.55\mathrm{\%}$ in case of $\upbeta =3.32$ from $12.64 \; \mathrm{ MPa}$ in case of unreinforced glulam model to $12.71 \; \mathrm{ MPa}$ in case of reinforced glulam model.

## Conclusions

In this study, probabilistic nonlinear finite element models were considered for analyzing reinforced glulam beams with CFRP plates and unreinforced glulam beams. Hill’s yield criterion model is utilized to validate the numerical model in which the results of experimental tests approved the numerical predictions. Furthermore, a written code including the adoption of reliability index as a factor that controls the analysis bound is utilized in which the timber properties are considered as random variables following a normal distribution with mean value and standard deviation.

Thus, as per what have been mentioned already, the concluded key points are:

- In both models, it was noticed that considering $\upbeta$ has influenced the results of corresponding loads $(\mathrm{F})$ and displacements $(\mathrm{U})$.
- For each model, the results show that as $\upbeta$ declines, the corresponding values of mean von mises stress increase.
- Due to the probabilistic nature of timber properties, the load $(\mathrm{F})$ and displacement $(\mathrm{U})$ values were directly affected for both cases of reinforced and unreinforced models.
- The pattern of normal stress distributions was less intensive in the case of probabilistic analysis than in the case of deterministic analysis, thus it can be said that $\upbeta$ works as a controlling limit that produces a safe design.
- The effects of considering the CFRP plate as reinforcement material of the glulam beams were noticeable according to the obtained results which are related to load $(\mathrm{F})$, displacement $(\mathrm{U})$ and mean von mises stress values for deterministic and probabilistic designs.
- There is a quite well match between the numerically obtained force–deflection diagrams with experimentally obtained diagrams. Consequently, the model can predict the nonlinear behavior of the unreinforced and the reinforced beams.
- It was approved that numerical modeling is effective in the bending behavior analysis of both unreinforced and reinforced beams, thus saving expected resources for experimental tests.

The work presented in this paper can be seen as a significant development into a more reasonable framework for the nonlinear probabilistic analysis of the reinforced glulam beams with CFRP plates. However, additional examinations and research works are supposed to consolidate other nonlinear issues such as fatigue damage and fracture.

## Data Availability

The whole datasets which are generated during and analyzed during the current study are available in the main manuscript.
